# ECG challenge: unexpected shift in QRS morphology

**DOI:** 10.1093/ehjcr/ytag157

**Published:** 2026-03-06

**Authors:** Pietro Redigolo, Paolo Zappulla, Davide Capodanno

**Affiliations:** Division of Cardiology, Azienda Ospedaliero-Universitaria Policlinico ‘Rodolico – San Marco’, University of Catania, Via Santa Sofia, Catania 78 - 95123, Italy; Division of Cardiology, Azienda Ospedaliero-Universitaria Policlinico ‘Rodolico – San Marco’, University of Catania, Via Santa Sofia, Catania 78 - 95123, Italy; Division of Cardiology, Azienda Ospedaliero-Universitaria Policlinico ‘Rodolico – San Marco’, University of Catania, Via Santa Sofia, Catania 78 - 95123, Italy

**Keywords:** ICD, Heart failure, Atrial fibrillation, Cardiac conduction disturbance

An 84-year-old male with a history of hypertension, type 2 diabetes mellitus, dyslipidaemia, atrial fibrillation and chronic kidney disease, first experienced the onset of exertional dyspnoea accompanied by palpitations in February 2025. Due to the persistence of these symptoms, a comprehensive diagnostic workup was performed in June 2025, revealing heart failure with reduced ejection fraction (HFrEF) of non-ischemic origin, as confirmed by the absence of obstructive coronary artery disease on invasive coronary angiography. Following the diagnosis, the patient was started on guideline-directed medical therapy (GDMT).

Despite 3 months of such management, a follow-up echocardiogram showed persistent systolic impairment with a left ventricular ejection fraction (LVEF) of 35%. Given the lack of substantial recovery and the risk of sudden cardiac death, the patient met the criteria for primary prevention. Consequently, he was admitted in January 2026 for elective implantation of an implantable cardioverter-defibrillator (ICD).

A pre-procedural ECG showed atrial fibrillation with a complete right bundle branch block (RBBB) pattern. During the routine post-procedural clinical monitoring in the inpatient ward, a 12-lead ECG was performed, capturing a spontaneous transition between RBBB and left bundle branch block (LBBB) morphologies within the same recording (*Figure 1*).

**Figure 1:**
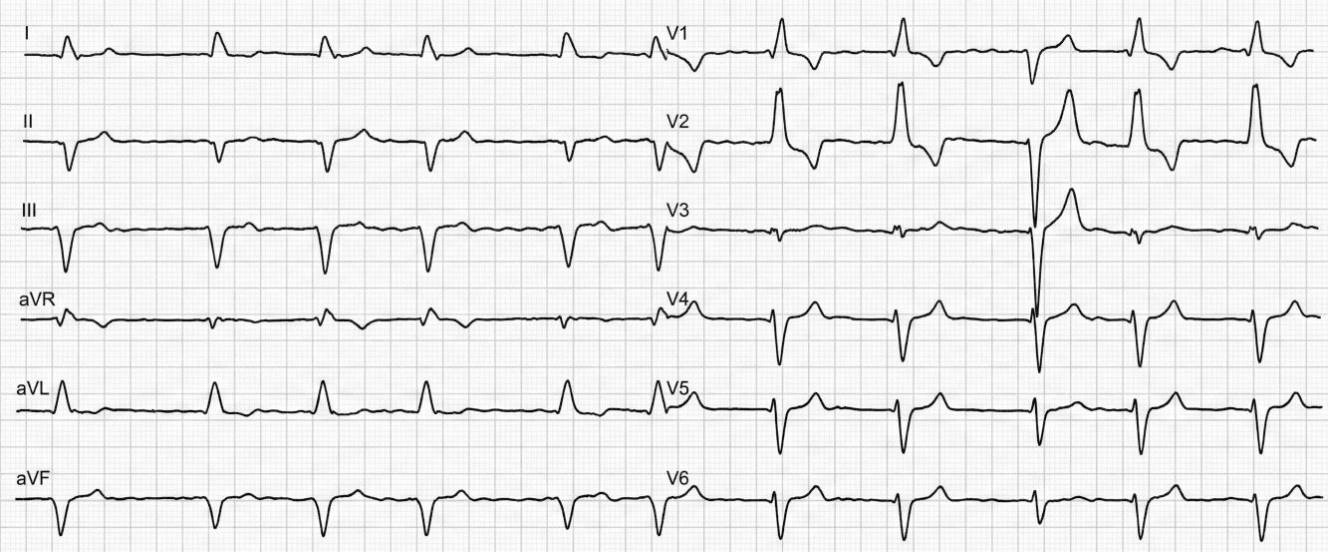
12-lead ECG performed one day after implantable cardioverter-defibrillator implantation.

## Question 1: What is the most likely diagnosis for the rhythm transition seen in *Figure 1*?

- Rate-dependent Ashman phenomenon.
- Alternating Bundle Branch Block (ABBB).
- Intermittent ventricular pacing.
- Intermittent pre-excitation.

Correct Answer: B

## Discussion and explanation

The transition seen in *Figure 1* is diagnostic of ABBB, defined as spontaneous alternation between RBBB and LBBB morphologies in the same patient. Unlike rate-dependent aberrancy (Ashman phenomenon), the alternation occurs without a consistent long-short RR sequence. Moreover, no pacing artefacts or pacing-related QRS morphology are evident, excluding intermittent ventricular pacing.

ABBB reflects advanced conduction system disease involving both bundle branches, indicating severe and unstable infra-Hisian conduction. This pattern is widely recognized as a marker of impending complete atrioventricular (AV) block rather than a functional or benign conduction abnormality.^1^

### Question 2: What is the primary clinical implication of this ECG finding?

- It represents a benign variant of intraventricular conduction delay.
- It suggests lead dislodgement or micro-dislodgement after ICD implantation.
- It indicates an imminent risk of progression to complete (third-degree) atrioventricular block.
- It is a pathognomonic sign of acute myocardial ischaemia in the septal area.

Correct Answer: C

## Discussion and explanation

From a clinical standpoint, recognition of ABBB is crucial, as it is associated with a high and unpredictable risk of progression to complete AV block and ventricular standstill, even in asymptomatic patients. Both European and American guidelines consider ABBB a Class I indication for permanent pacing, owing to the advanced infra-Hisian nature of the conduction disease.^2,3^

In this specific case, the recently implanted ICD already provides the necessary backup pacing; although no immediate device upgrade is required, the presence of ABBB underscores the need for careful follow-up, as it may precede future pacing dependency or the development of conduction patterns relevant to resynchronization strategies.^2,3^

### Question 3: What is the most likely anatomical site of the conduction delay in a patient exhibiting alternating bundle branch block?

- Proximal AV node.
- Intranodal AV node.
- Infra-Hisian conduction system.
- Left posterior fascicle with compensatory right-sided delay.

Correct Answer: C

## Discussion and explanation

Alternating bundle branch block is a hallmark of advanced infra-Hisian conduction system disease, typically involving bilateral bundle branch pathology. Unlike AV nodal disease, which often manifests as Mobitz I AV block, ABBB reflects severe conduction instability distal to the His bundle, with unpredictable propagation through the His–Purkinje system. Electrophysiological studies in such patients frequently demonstrate markedly prolonged HV intervals and confirm a high likelihood of progression to complete AV block or ventricular standstill.^1^

## Data Availability

All data are available upon reasonable request to the corresponding author.

## Notes

**Consent:** Direct written consent was obtained from the patient for the publication of this manuscript, in accordance with Committee of Publication Ethics (COPE) guidelines.
